# 

**Published:** 1839-01-05

**Authors:** 


### CONTENTS OF VOLUME II.

Abernethiana, 351.
Abscess, Dr. Graves' case of abdominal, 177 : of the lung, 500.
Absorption, cutaneous, Dr. Madden on, bibliographical notice of, 557.
Algiers, state of medicine in, 224.
Alumina, sulphate of, to preserve bodies for dissection, 196.
Amaurosis, treatment of, 692 : case of, 794 : Dr. Colhoun's case of, 806.
Amputations, statistics of, 173, 537 : at the shoulder-joint, 324, 601 : Mr. Liston on, 337.
Andral, professor, appointment of, 128.
Aneurism, case of, 115, 457.
Anus, artificial, cases of, 564, 592, 820.
Areola, the mammary, 227.
Aorta, case of wound of, 148.
Aphonia, cure of, 628.
Arsenic, case of poisoning by, 146 : in uterine affections, 164 : combination of, with antimony and hydrogen, 226 : Dr. Vernois' letter on, 454.
Ascites, Dr. Stewardson's case of, 773.
Association, proposed scientific, 32 : British Medical, proceedings of, 722.
Astley Cooper's surgery, bibliographical notice of, 558.
Autoplasty after cancerous excisions, 675.
Autopsy of lady F. Hastings, 498.

Basilar artery, Dr. Davy on, 179.
Bed-sores, new treatment for, 228.
Bedford, Dr., notice of introductory lecture by, 698.
Betton, Dr. T. F.'s case of strangulated hernia, 245 : case of dislocation of os femoris, 629 : on carbuncle, 807.
Billard on the diseases of infants, bibliographical notice of, 747.
Black drop, 813.
Bladder, irritable, 352.
Blennorrhœa, in the teething of children, 643.
Blistered surfaces, application for, 212.
Blood, sugar in the, 20 : globules of, in the camel and alpacha, 340 : M. Magendie on, 499 : M. Andral on coagulation of, 628 : on the changes of, in fevers, 640.
Boerhave, sketch of, 579.
Bones, Nelaton on tubercular disease of, 476.
Breast, Dr. J. F. Meigs' case of tumour of, 711.
Bronchi, case of foreign body in the, 228.
Bronchocele, formula for, 68.
Broussais, professor, death, character and autopsy of, 29, 33, 68.
Buchu, notice of, 19.
Burdock, in impetigo, 68.
Burns, treacle as a dressing for, 65 : treatment of cicatrices from, 212 : Mr. S. Cooper on, 242.
Burns' Surgery, bibliographical notice of, 59.

Cadogan, Dr., sketch of, 708.
Calculi, case of, 350.
Camphor in diseases of the respiratory apparatus, 500.
Cancer, old mode of operating in, 596 : of the foot, Dr. Stillé's case of, 646 : of the stomach, case of, 789.
Cancrum oris, Dr. Webb on, 549.
Carbonic acid in phthisis, 100.
Carbuncle, caustic potash in first stage of, 807.
Carditis, case of, 128.
Carson, professor, lecture by, on materia medica, 360 : on pharmacy, 415.
Cataract, lecture on 62 : case of, 65.
Cauterization of the pharynx, 33.
Chapman, professor, lectures by, on hæmorrhage, 5, 21 : on hæmoptysis, 53, 69, 85, 101 : on epistaxis, 117, 133 : on hæmatemesis, 149, 165 : on menorrhagia, 197, 213 : on hæmaturia, 229.
Chauncey, trial of, for murder, 72, 78, 292.
Chilblains, Dr. Graves on, 36 : application for, 692.
Chlorosis, M. Trousseau on, 611.
Circocele, Mr. Liston on, 338.
Cirrhosis, clinical lecture on, 544.
Cisampeline, 388.
Clarke, Sir C. M., sketch of, 84.
Clavicle, case of fracture of, 55.
Clergymen, cause of ill-health of, 347.
Clitoris, lecture on, 314.
Clymer, Dr., on suicide from suspension, 24.
Coates, Dr. B. H., lecture by, on delirium tremens, 31 : letter from, 78.
Colchicum, the external use of, in rheumatism, 301.
College, Philadelphia Medical, 140, 497, 699.
Congestive fever, Dr. Buck on, 599 : Dr. Malone on, 714.
Copaiba, syrup of, 675.
Coroners, medical, 212, 651.
Corpus luteum, remarks on, 94.

Corrosive sublimate, case of poisoning from, 189.
Counter-stimulation, Dr. Martins' letter on, 342.
Coxalgia, Dr. W. Harris on, 37.
Creosote, in deafness, 34: Dr. I. Parrish on, 501: styptic action of, 644.
Croup, Dr. Reiersen on, 88.

Daguerrotype, account of, 602, 673.
Delirium tremens, lectures on, 31, 797, 807: Dr. Klapp on, 58, 90: Dr. Mendenhall on, 77: Dr. Coates on, 78: Stoll on, 150.
Denis' physiological essay, Dr. Vernois' letter on, 474.
Denny, Dr. W. case of difficult labour by, 10.
Desquamation, extensive, 36.
Diseases of Philadelphia in 1839, 539.
Dislocation of the humerus, 34, 590: of the astragalus, 67: old, 83: of the os femoris, 629, 639, 803: of the patella, 653.
Dispensary, Philadelphia, reports from, 11, 43, 44, 118, 231, 349, 394, 453, 473, 664: Dr. Warrington's lecture on, 421.
Dispensatory, U. S., new edition of, 372.
Doane, professor, election of, 14.
Doses of active remedies, 468.
Dublin School of Medicine, letter from Dr. Clymer on, 536.
Dumas, professor, 372.
Dunglison's Medical Dictionary, bibliographical notice of, 433.
Dupuytren's pommade for the hair, 592.
Dysentery, Dr. Wooten on, 437: Dr. Griscom on, 565: lectures on, 749, 781.

Ear, lectures on the diseases of, 108, 123, 141.
Eczema impetiginodes, lecture on, 231.
Education, medical, 699.
Eighth pair of nerves, Dr. Reid on, 607.
Empyema, case of, 606.
Endocarditis, Dr. Kerr's case of, 805.
Epidemics of United States, 779.
Epilepsy cured by acupuncture, 644.
Episioraphy, cases of, 420.
Epistaxis, Dr. Graves on, 35: lectures on, 117, 133.
Ergot of rye, Mr. Quekett on, 530, 540: its effects on the fœtus, 836.
Erysipelas, M. Blandin on, 147: Desault's treatment of, 308; belladonna in, 404: raw cotton in, 457.
Esophagus, case of foreign body in, 113.
Eustachian tube, death from pumping air into, 548.
Excretory ducts, muscular coats of, 788.
Exostosis of the pelvis, case of, 223.
Eye, Dr. Wallace on, bibliographical notice of, 508: Dr. M'Clellan's case of extirpation of, 600.

Fairfax, Dr. O.'s case of difficult labour, 826.
Feeding of infants artificially, 420.
Femur, lecture on osteitis of, 464.
Fever, Dr. Thomson on, bibliographical notice of, 172: Dr. Shook on, bibliographical notice of, 505: Dr. Lendrick on, 514, 523: petechial of St. Petersburg, 675: of Chicago, Dr. Brainard on, 715.
Fœtus, account of a double-headed, 330: venereal contamination of, 436.
Fire arms, means of ascertaining when discharged, 268.
Five children at a birth, case of, 804.
Fractures, on the treatment of the immoveable apparatus, 143, 148, 221, 803: of the coracoid process of the scapula, 210: of the skull, 214, 251, 577: of the thigh, 250: of the ribs, M. Malgaigne on, 384: ununited, 652: on reparation after, 139.
Frog, on the cerebral nerves of, 207.
Frost-bite, lecture on, 142.
Fungoid tumor of the tibia, Dr. Stillé's case of, 665.

Ganglionic system, Dr. Reid on, 635.
Gangrene, Dr. Zollickoffer's case of, 169: of the penis, Dr. Rivers' case of, 577; lecture on chronic, 761.
Generation, equivocal, 690.
Gentianine, 388.
Gerhard, Dr., lectures by, on pneumonia, 81, 106, 216, 235, 293: on tuberculous disease, 446, 508, 631: on phthisis, 781, 797, 807, 827: on intermittent fever and pneumothorax, 761: on dysentery, 749, 781: on delirium tremens, and gangrene of the lungs, 797, 807.
Gibson, professor, lectures by, on burns, staphyloma, cancroides, 16: on hare-lip, 761, 807: on chronic gangrene, 761: on osteo-sarcoma, 785: sketches by, of London and Parisian surgeons, 725.
Glanders in man, 436, 643.
Gonorrhœa, Dr. Graves on, 97.
Gravel, M. Civiale on, 690.

Hospitals, Pennsylvania, reports from, 18, 65, 94, 173, 250, 511, 539, 576: Philadelphia, reports from, 152, 181: Massachusetts, report from, 189: Wills', report from, 233: of Holland, 244: Dublin reports, 244: La Charité of Paris, 605, 672: Hôtel Dieu of Paris, 672: Ophthalmic at Canton, 712.
Hot Springs of Virginia, 415.
Hunter, new edition of works of, 212: sketch of, 336.
Hydrocele, iodine in, 99.
Hymen, Dr. Latta's case of imperforate, 677.
Hypertrophy of the colon, Dr. Thomas' case of, 585: of the heart, Dr. Caspar Morris' case of, 795.
Hæmatemesis, lectures on, 149, 165.
Hæmaturia, lecture on, 229.
Hæmoptysis, lectures on, 53, 69, 85, 101.
Hæmorrhage, lectures on, 5, 21: Dr. Strobhart's case of, 312: iodide of iron in, 324.
Hall, Dr. M.'s, works, bibliographical notices of, 105, 680: lectures by, 130: Mr. Liston's case of, 339.
Harlan, Dr., letters from, 119, 134, 377, 440, 453, 488, 613.
Harris, Dr. T., lectures by, on cataract, 62: on pterygium, 65: on the lachrymal duct, 121.
Harris, Dr. W., cases of preternatural labour by, 9: surgical cases by, 26: on coxalgia, 37:

case of hydrocele by, 41: case of fracture of vertebra by, 70: lectures by, on the clitoris, 314, on prolapsus uteri, 326, on retroversio uteri, 347, on anteversio uteri, 382, on leucorrhœa, 394.
Heart, lectures on the diseases of, 459: Bouillaud on coagula in the, 494: Dr. Pennock on the normal condition of, 533: Dr. Stillé's case of dilatation of, 581: Drs. Pennock and Moore's experiments on the action of, 693: on coagula in, 706.
Heberden, sketch of, 580.
Hepatic abscess, Dr. Graves on, 16.
Hepatitis, lecture on, 397.
Hernia, cases of strangulated, 65, 245, 287, 741: M. Gerdy's plan for the radical cure of, 647.
Hildreth, Dr., address by, 424.
Hip, on the diseases of, 707.
Hippocrates, new edition of works of, 667.
Hooping-cough, subcarbonate of iron in, 36.
Horner, Dr. G. R. B.'s work on the Mediterranean, bibliographical notice of, 587.
Horner, professor, lectures by, on the ear, 108, 123, 141: on frost-bite, 142.

Ichthyosis, Dr. Neil's case of, 440.
Infanticide, case of, 205.
Infection and contagion, Dr. Beaugrand's letter on, 552.
Inflammation, M. Magendie on, 295.
Influenza, at Lisbon, 100.
Infusoria, professor Grant on the, 239.
Insanity, Dr. Cottman's case of, 583: influence of civilization on, 592.
Interments of New York, 217.
Intermittent fever, lecture on, 764.
Iron, on the carbonate and protomuriate of, 113: formula for, 676: as an antidote for arsenic, 787.

Jackson, professor, lecture by, on scrofula, 46.
Jalap, substitute for, 740.
Johnson, Dr. James, sketch of, 345.
Journals, Louisville Medical, 203: New York Medical, 284, 445: of the Medical Society of New Orleans, 345: Western, 632.

Keloides, Dr. Kerr's case of, 680.
Kidneys, Sir B. Brodie on disease of, 319.
Kisteine, in the urine of pregnant women, 804.

Lachrymal duct, lecture on, 121.
Leucorrhœa, lecture on, 394.
Liquor amnii, analysis of, 52.
Lithophagus, history of a, 674.
Lithotomy, statistics of, 112, 196: lecture on, 201.
Lithotrity, antiquity of, 151, 201.
Liver, case of absence of, 611.
Lumbrici in the peritoneum, Dr. Thomas' case of, 77.
Lunatic Asylum, of the Philadelphia Hospital, report from, 56: of Massachusetts, 175.
Lyttæ, the external use of in rheumatism, 301.

Malformations of the viscera, 212.
Membranes, the mucous and cutaneous, 211.
Meningitis, Dr. Halson's case of, 678.
Menorrhagia, lectures on, 197, 213.
Menstruation, Dr. Miller's case of vicarious, 518, 586.
Milk sickness, 512.
Miner, Dr. T.'s introductory lecture, bibliographical notice of, 187.
Mineral waters, account of the French, 325, 373, 389, 405.
Mitchell, Dr. T. D.'s introductory lecture, bibliographical notice of, 187.
Morphia, the endemic application of, 290.
Mütter, Dr., on club-foot, bibliographical notice of, 170.

Nævus, nature and treatment of, 269.
Nerves, M. Gerdy on the sensitive and motor, 361: Dr. Beaugrand's letter on, 502.
Neuralgia, of the testicle, Dr. Graves on, 178: of the larynx, 180: mixture for, 468.
Norris, Dr., Liston's Surgery by, bibliographical notice of, 139

Ohio, proceedings of medical convention of, 415.
Ophthalmia, Mr. Arnott's lecture on, 321: Mr. Carmichael on purulent, 433: Dr. Webb on purulent, 485: Velpeau's lectures on, 603, 624, 633, 655, 668, 681, 684, 700, 717, 752, 767, 814, 832.
Orthopœdic establishments, Dieffenbach on, 351.
Osteo-sarcoma, Dr. Andrews' case of, 168: lecture on, 785.
Ovarian abscess, Dr. Patterson's case of, 104: tumour, Dr. Bush's case of, 662.

Paralysis, lecture on, 316.
Paraplegia, Mr. Key on, 771.
Parotid gland, extirpation of, 14, 27, 66.
Parsons, Dr. U. S.'s Boylston Prize Dissertations, bibliographical notice of, 249.
Partus per anum, case of, 116: Dr. Andrews' case of, 169.
Patents, resolution touching, 284.
Pathological Societies, of Dublin, 150, 642: of Philadelphia, 632.
Pattison, professor, notice of introductory lecture by, 12.
Pennock Dr., case by, 469, 533: experiments by, 693.
Pericarditis, Dr. Graves on, 253.
Peritonitis, lecture on, 450.
Phthisis pulmonalis, lectures on, 416, 781, 797, 807, 827: relative frequency of, 483.
Physick, Dr. Randolph's memoir of, 261.
Physiology, Dr. Carpenter's, bibliographical notice of, 233.
Pins and needles, on the swallowing of, 219.
Pituitary gland, 336.
Pleurisy, Dr. Graves on hæmorrhagic, 194.
Pneumonia, lectures on, 81, 106, 216, 235, 293.
Pneumothorax, case of, 145: lecture on, 764.
Poisoning from vapours of charcoal, 769.
Polypi of the pharynx, 204.
Pork, Dr. Dupuy's case of poisoning from, 66.
Porrigo, pathology of, 356: clinical lecture on, 558.

Pregnancy, extra-uterine, cases of, 49, 452, 760: Dr. Meigs' case of, 709.
Professional charges, 315, 497.
Pterygium, lecture on, 65.
Pulmonary artery, case of obliteration of, 260.
Purgatives, Dr. Beaugrand's letter on, 519.
Pus, researches on, 128.

Quinine, adulteration of, 35: in yellow fever, 666, 681: citrate of, 674: Dr. Donoho on the use of, in malignant fever, 760.

Radesyga, Dr. Martins' letter on, 422.
Randolph, Dr., lectures by, on the parotid gland, 14: on lithotomy, 201: memoir of Dr. Physick by, 261.
Rectum, M. Liston on prolapsus of, 338.
Remittent fever, Dr. Burns on, 597: Dr. Mendenhall on, 600.
Re-vaccination, Dr. F. C. Stewart on, 200: statistics of, 612.
Rheumatism, lectures on, 367, 478: Dr. Watkins on the use of aconite in, 517.
Ricord, M. on phimosis, 596.
Rotatores dorsi, discovery of, 564.
Rue, in abortion, 302.
Rush, Dr. B.'s essay, bibliographical notice of, 126.
Rupture of the vena cava, 19: of the bladder, 203.

Saratoga waters, 252.
Scarlatina, Dr. Cross on, bibliographical notice of, 138: Dr. Keckeley on, 458: Dr. Glizen on, 742: Dr. Borland on, 825.
Schools, increase of medical, 780.
Scirrhus of the esophagus, Dr. Graves on, 240: of the lung and heart, 260.
Scrofula, lecture on, 46: on the treatment of 256: on the use of gold in, 308.
Silver, on the use of, in syphilis, 743.
Sinapisms, Dr. Graves on, 180.
Skin, case of detachment of, 114.
Snake-man, account of, 176.
Sœurs de la Charité, 355.
Societies, Philadelphia Medical, 10, English Medical at Paris, 260.
Spinal marrow, Dr. Graves on inflammation of, 178: case of inflammation of, 341.
Spine, Dr. Guérin on deviations of, 594.
Statistics of W. India troops, bibliographical notice of, 59, of St. Petersburg, 340.
Strangulation, on suicidal, 24, 206, 788.
Sudden death, Dr. Ollivier on, 204.
Syphilis, Dr. Graves on, 284, 304.

Taraxacum, extract of, 243.
Tracheotomy, statistics of, 836.
Temperature, the influence of in healing wounds, 227.
Tendons, on the cicatrization of, 260.
Thermometers, Dr. Martins' letter on, 413.
Thorax, Dr. Tomlinson's case of wound of, 76.
Tinea capitis, ointment for, 116: Dr. Zollickoffer's formula for, 199: Sir B. Brodie on, 484.
Tonsils, enlarged, Dr. Graves on, 195: Dr. J. M. Warren on, 309: Sir B. Brodie on the use of iodine in, 310.
Trachea, Dr. Sharpless' case of compression of, 57.
Triticum and ægilops, 820.
Tuberculous disease, lectures on, 446, 508, 631.
Tumours, on the removal of, by deligation, 463.
Turpentine, formula for, 692.
Typhoid fever, Dr. Hale on, bibliographical notice of, 629: Dr. Miller's case of, 761.
Typhus, Dr. West on, bibliographical notice of, 12: Dr. Graves on, 50: Dr. Stokes on, bibliographical notice of, 245; Dr. Roupell on, bibliographical notice of, 505: the saline treatment in, 590.

Ulcers, Dr. Graves on, 49: of the stomach, M. Cruveilhier on, 192.
Universities of New York, 111: of London, 130: Transylvania, 188: Jefferson, 189, 449, 632: of Berlin, 209: of Pennsylvania, 233: Albany, 457, 589, 624: of Louisville, 601.
Urine, albuminous, 693.
Uterus, amputation of the neck of, 36: child expelled from, after interment, 42, 316: Dr. Kennedy on hypertrophy of the mouth of, bibliographical notice of, 44: case of spontaneous inversion of, 83: case of fibrous tumour of, 320: lecture on prolapsus of, 326: lecture on retroversion of, 347: lecture on anteversion of, 382: case of abstraction of, 449: case of fibro-scirrhous tumour of, 469: on excoriations of the neck of, 675: rheumatism of, 688: Dr. Stewardson's case of tumour of, 757: removal of fibrous tumour of, 802.

Vagina, on the temperature of, 404.
Valleix on the diseases of infants, bibliographical notice of, 157.
Varicocele, M. Landouzy on, 594.
Varix, fatal case of, 791: Dr. H. H. Smith on, 821.
Veratria in rheumatism, Dr. Latta on, 104.
Veratrium, Dr. Adams on, 55.
Vertebra, fracture of, 70.
Vertebræ, Sir B. Brodie on disease of, 302.
Vomit, black, Dr. Robertson on, 661.
Vulva, Dr. Wright's case of polypus of, 169.

Weevil, Dr. Ridley on the vesicating properties of, 743.
Williamson, Dr. Hugh, on the health of armies, 41.
Wistar's Anatomy, Dr. Pancoast's edition of, bibliographical notice of, 93.
Wry-neck, on the division of the sterno-cleido-mastoid in, 403, 459.

Yellow fever in Texas, 697: on the contagious character of, 703: M. Louis on, bibliographical notice of, 775.

Zinc, gray oxide of, 388.

